# Quality of life and well-being from the perspective of patients on opioid agonist maintenance treatment: study protocol for a systematic review of qualitative research and a scoping review of measures

**DOI:** 10.1186/s13643-019-1237-8

**Published:** 2019-12-01

**Authors:** Ivan Solà, Joan Trujols, Elisa Ribalta, Saul Alcaraz, Gemma Robleda, Clara Selva Olid, José Pérez de los Cobos

**Affiliations:** 1 Iberoamerican Cochrane Center, Biomedical Research Institute Sant Pau (IIB Sant Pau), Barcelona, Spain; 2 CIBER Epidemiología y Salud Pública (CIBERESP), Madrid, Spain; 3 Addictive Behaviours Unit, Department of Psychiatry, Hospital de la Santa Creu i Sant Pau, Barcelona, Spain; 4 Biomedical Research Networking Center on Mental Health (CIBERSAM), Barcelona, Spain; 5 Addictive Behaviors Research Group, Biomedical Research Institute Sant Pau (IIB Sant Pau), Barcelona, Spain; 6 Escuela Superior de Enfermería Mar, Barcelona, Spain; 7 Departament de Ciències Experimentals i de la Salut, UPF, Barcelona, Spain; 8 PETRO Research Group, Bellaterra, Spain; 9 Department of Social Psychology, Universitat Autònoma de Barcelona (UAB), Bellaterra, Spain; 10 Department of Psychiatry and Forensic Medicine, School of Medicine, Autonomous University of Barcelona (UAB), Bellaterra, Bellaterra, Spain

**Keywords:** Opioid maintenance treatment, Quality of life, Well-being, First-person perspective, Qualitative research, Personomics, Patient-centered research

## Abstract

**Background:**

Opioid agonist maintenance treatment (OAMT) is a first-line treatment for heroin dependence, but its effectiveness has been assessed primarily through clinical outcomes with a limited attention to patient perspectives. Despite the increased use of patient reported outcome measures their patient-centeredness is highly questionable. This is the protocol of a systematic review of qualitative research on how OAMT users construct the meaning of their quality of life and well-being and a scoping review of instruments that measure these domains.

**Methods:**

We will conduct a systematic review of qualitative research exploring the views of quality of life of patients on OAMT (registration number CRD42018086490). According pre-specified eligibility criteria, we will include studies from a comprehensive search of bibliographical databases from their inception. We will extract data from included studies and assess their risk of bias with the CASP appraisal criteria, and will implement a thematic analysis to generate a set of interpretative analytical themes ascertaining their confidence using the CERQual approach. We will implement similar methods to conduct a scoping review to assess to what extent the existing measures of these domains were focused on user’s views, assessing their validity using the COSMIN methodology, and summarizing their characteristics and level of patient centeredness.

**Conclusion:**

The findings from the reviews will contribute to obtain a genuine understanding of the perspective from users on OAMT regarding their perception of well-being and quality of life and will likely lead to greater patient centeredness when assessing such variables, which in turn may contribute to a more patient-centered care.

## Background

Nowadays opioid agonist maintenance treatment (OAMT) is a first-line psychopharmacological treatment for heroin dependence [1]. Numerous studies have consistently shown that OAMT is an effective treatment for opioid dependence [2–6]. However, the effectiveness of OAMT has been assessed primarily—indeed, almost exclusively—through clinical outcomes or “hard” indicators [7], such as treatment retention, reduction of heroin or other non-prescribed opioids use, a reduction of morbidity and mortality, and decreases in criminality, among others. While these outcomes and indicators are valid and should be assessed—especially in efficacy studies—they are often used to justify health care policies or to seek community approval. Moreover, such indicators are based on a negative and disorder-centered conception of health [7] (see Additional file 1 Table S1).

The selection and assessment of the outcomes used to evaluate the effect of interventions (such as OAMT) in the field of addictive disorders remains challenging, not only in the setting of clinical trials but particularly in routine clinical practice [8–10]. Nevertheless, the role of the patient perspective in such selection process has received little attention. At present, assessment of OAMT efficacy and effectiveness primarily focuses on outcomes defined by the scientific and clinical community, and these variables may not adequately reflect the patient’s perspective, or they may not assess the outcomes in a way that is relevant to them.

The most developed line of research in exploring the OAMT patient’s perspective is focused on quality of life or, in a broader approach, on the well-being construct, since both terms are often used interchangeably [11, 12].

A growing number of authors, both in the field of addictive disorders [13, 14] and in other areas of health care [15, 16], have questioned whether the scales most commonly used to assess QoL or well-being actually provide a truly patient-centered evaluation of this construct. In this respect, De Maeyer and collaborators explored the nature and dimensions of this construct from the perspective of patients receiving treatment for opioid dependence [17]. The study showed that patients do not primarily associate QOL with health, and moreover, they spontaneously reported many more issues than those usually included in the instruments commonly used to assess QoL in these patients. Similarly, a recent QOL instrument developed with significant input from OAMT patients did not include items on physical health [18].

In fact, in certain areas of health outcomes research [19–21], there seems to be a growing consensus that patients should be significantly involved—through truly participatory methods—in developing any new patient-reported outcome measure (PROM). The development of new PROMs, however, should not be promoted without synthetizing the existing knowledge allowing its application to practice and the process of decision-making and avoiding the production of research of little value [22–24]. However, to date—and despite a certain shift in how OAMT results are evaluated, with a greater emphasis on including patient perspectives—we still lack accurate reviews to evaluate the extent to which patient perspectives have been incorporated into the development of instruments evaluating QoL and well-being in patients on OAMT and to obtain an integrated and interpretative representation of how these meanings are constructed from the OAMT patient’s perspective. The application of knowledge to action framework should allow to implement such syntheses of the existing knowledge in the elaboration of QoL and well-being assessment tools genuinely generated by patients [24, 25].

Given this context, here, we propose two interrelated studies whose main aims are to, at least partially, address the knowledge gap in this area and to remedy the paucity of patient-centered insight to assess QoL and well-being in patients on OAMT. Our aim is to conduct a systematic review of qualitative research on how OAMT users perceive, experience and construct their quality of life and well-being and a scoping review of instruments that measure this domain.

## Methods

We selected two specific methods for conducting knowledge synthesis adapted to the scope and nature of the topic of interest (how quality of life (QoL) and well-being have been defined from OAMT users’ views), which are described below. The systematic review of qualitative research will allow us to increase understanding of the phenomenon of interest and its complexity [26], and the scoping review will explore the extent, range, and characteristics of existing measures for the aforementioned domains [27]. We adhered the PRISMA-P guideline to report this protocol (provided in the Additional file 2).

### Study 1: Systematic review of qualitative research

We will conduct a systematic review of qualitative research exploring OAMT users’ views and perspectives on QoL to synthesize them into an integrated and interpretative representation [28]. We have prepared a protocol following methodological standards [29] and according to specific guidance to synthesize qualitative research [26, 30, 31], that was publicly registered in PROSPERO [32] (registration number CRD42018086490). We will report the review findings endorsing the ENTREQ reporting guidelines [33].

#### Inclusion criteria

The review will include studies focusing on individuals diagnosed with opioid dependence disorder (according to the current version of either the Diagnostic and Statistical Manual of Mental Disorders (DSM) or International Classification of Diseases and Related Health Problems (ICD) diagnostic systems) and who are receiving – or have recently received – OAMT. Eligible studies will focus on their views, experiences and perspectives about the construct of interest, considering those studies that have used the phenomenological perspective of a qualitative research.

#### Search methods to identify studies

We will perform a search in MEDLINE (Ovid), EMBASE (Ovid), CINAHL (EBSCOHost), PsycINFO (Ovid), and Cork database (Project Cork) from their inception. We will design a search strategy combining terms related to the inclusion criteria as well as controlled vocabulary related to these terms [for the strategy designed to search MEDLINE see Additional file 3]. The search algorithms will be adapted to the specific requirements of each specific database. We will execute the searches in these databases with no limitations other than language (restricted to English, French, German, Dutch and Spanish) and the use of validated filters to obtain qualitative research [34–37].

In addition to the search in databases we will identify studies from the reference lists from the studies initially included in our review, and will search unpublished studies in Open Access Theses and Dissertations, ProQuest Dissertations & Theses Database and through a specific search in Google Scholar. Besides, we will track references of additional studies citing the eligible studies using the Web of Science.

#### Study selection and data collection

We will manage the search results using a reference manager software. Two researchers will independently screen the references’ titles and abstracts against the inclusion criteria, obtaining a copy of eligible studies to determine their final inclusion. We will describe the entire eligibility and selection process into a PRISMA flowchart and report the reasons for exclusion of ineligible studies in a specific table.

Although the optimal approach to identifying sources of bias in a qualitative study is a matter of discussion [38–40], there is guidance to use approaches focusing at least on the clarity of the study aims and its research question, the congruence between aims and research design and methodology, the rigour in sampling and case identification, and the appropriateness of the application of the method, conceptual depth of findings, exploration of deviant cases, and reflexivity of the researchers [41]. The Critical Appraisal Skills Programme (CASP) appraisal criteria are the most commonly used for this purpose and so we will use these criteria to assess the studies included in the systematic review of qualitative research [42].

We will extract data in two stages. First, we will extract study design and descriptive information and then, we will extract quotes and categories identified through the included studies' findings. Data collection in this second part will be iterative, and we will revisit the texts to compare ideas and original findings in light of any new data identified [43–45].

We will pilot a pre-defined data extraction form appraising at least three studies to verify the structure of the form and the suitability of the process.

#### Data analysis and synthesis of findings

We will use an integrative and interpretative approach for the findings from the systematic review of qualitative research through a continuous comparison of results from the original studies, implementing a thematic analysis standardized methodology [44, 45]. Based on the data collected from the studies, we will code emerging themes and iteratively compare them to recurring or new themes identified in other studies. Through the continuous comparison of data we will revisit previously reviewed studies to delimit potential thematic overlap and develop descriptive themes to finally generate contextualized, reflective and trustworthy analytical themes [43–45].

#### Assessment of confidence in the findings of the review

We will use the Confidence in the Evidence from Reviews of Qualitative research (CERQual) approach as a structured and explicit method to ascertain the confidence level that can be placed in the findings from the systematic review of qualitative research [46]. For each of the findings obtained in the thematic analysis, we will assess the following determinants of their confidence: methodological limitations; relevance (of the findings in the context of the research question); coherence (of the data to substantiate the explanation of phenomenon) and adequacy (of the data in terms of their quantity and richness in supporting the finding). Depending on the evaluation of each of these domains, the confidence level for each finding will be classified as high, moderate, low, or very low. A summary table will provide the findings of the review, the explanation or interpretation of those findings, the confidence classification, and an explanation of the criteria used to determine this classification.

### Study 2: Scoping review of measuring instruments of quality of life

In addition to the systematic review of qualitative research we will also conduct a scoping review to determine how and to what extent the patients' perspective has been incorporated into the development process of the measures used to evaluate QoL and well-being in patients on OAMT. We prepared a protocol according to standardized guidance [47], which is publicly available at Open Science Framework (https://osf.io/2cks9/). We will conduct the scoping review in accordance to the methodological framework proposed by Arksey and O'Malley to conduct a scoping review [47–49]. We will report the results of the scoping review according the corresponding PRISMA extension [27].

#### Inclusion criteria

We will consider for inclusion psychometric and /or development studies of instruments measuring QoL or well-being in patients which have been diagnosed with opioid dependence disorder according accepted diagnostic classification criteria and had been receiving OAMT.

#### Search methods to identify studies

We will conduct searches according to the methodology described for the systematic review of qualitative research [see Additional file 3 for the detailed MEDLINE search strings] but using a validated filter to obtain PROMs [50].

#### Study selection and data collection

After exporting search results to a reference manager software, two researchers will independently screen titles and abstracts and obtain copies of eligible studies to decide their inclusion in a process that will be detailed into a PRISMA flowchart.

We will design a data extraction form to collect study’s descriptive data and instruments’ characteristics according to: (a) the instrument type [OAMT specific, focused on the treatment of addictive disorders, or generic]; (b) its content validity, relevance, comprehensiveness and comprehensibility assessed using the COnsensus-based Standards for the selection of health Measurement INstruments (COSMIN) methodology [51]; (c) factor structure [global or unidimensional measures, or multidimensional instruments]; and (d) the extent to which patient perspectives are incorporated into the instrument, based on the level of patient involvement. We will assess patient involvement (i.e., consultation, contribution, collaboration, or control [52]) during all the stages of the tool's development: (a) conceptualization of the construct to be measured and determination of the domains to be assessed; (b) item generation; (c) evaluation of item relevance, comprehensiveness and comprehensibility; and (d) psychometric analysis.

#### Data analysis and synthesis of findings

We will integrate the characteristics included instruments, their content validity, the degree of patient centeredness, and the domains assessed following a thematic analysis framework [44], mapping the findings in accordance with the study objectives [47].

#### Utility and applicability of the findings

Once the findings of the scoping review have been obtained, a consultation process will be performed [48, 49] in which patients receiving OAMT will be contacted and asked to provide their views of these findings.

## Discussion

In this paper we describe the protocol of a systematic review of qualitative research to obtain a genuine understanding of the perspective from users on opioid agonist maintenance treatments (OAMT) regarding their perception of well-being and quality of life (QoL), as well as a scoping review to assess to what extent the existing measures of these domains were focused on user’s views. Applying a knowledge transfer framework [24, 25], the findings from these reviews will inform the drafting of the items of a patient-centered QoL scale that will be constructed according to measures development standards [53, 54]

The improvement and evaluation of processes and outcomes of care should be open to a broad approach that may reflect patient’s voice and perspectives. It has been hypothesized that patient reported outcomes (PROMs) improve patient’s skills at identifying, bringing meaning and describing health states. Shifting discussion into patients’ perspectives improves their functioning because it addresses some of their fundamental needs, enables them at constructing messages about their health state, and allows healthcare professionals to improve their understanding about these health states [55].

The measurement of relevant outcomes entails the challenge to evaluate processes of care focusing beyond the effectiveness of a determinate intervention, and broadening the perspective to the patient perception of its benefits or the response to treatment. PROMs have shown some positive impact in processes of care such as diagnosis, monitoring progression of disease or management of health conditions and response to treatment [56, 57]. These findings are specifically consistent in the field of mental health [58]. PROMs also constitute a presumably effective method to enhance the communication at medical encounter [59].

On the other hand, PROMs face some practical challenges hindering their ability to truly focus on patient perspective: who decides what to include and what to emphasize in such measures or, in other words, whether they reflect a true representation of patient experience [57]. Despite their potential content validity and relevance for respondents many PROMs have been developed without direct patient participation thus threatening the instruments’ capacity to be sensible to patients’ needs, priorities and preferences [13, 58]. For these reasons the development of measures that are truly representative of patient’s views and experiences should entail an understanding and capacity to capture their nuances and complexity.

Grounded in the knowledge to action framework [25], we plan to conduct a study to adapt and tailor the existing research on the views of patients that are on OAMT about QoL and well-being and implement this knowledge in the development of a patient-centered instrument. To ease the implementation of existing knowledge we are convinced of the role of syntheses of qualitative research in achieving a better understanding of complexity and subjectivity [28, 31]. The scoping review will also contribute to identify the key approaches already existing in the literature to the domain of interest and to define gaps in this area. For that reason we will conduct a review of qualitative studies to obtain a contextualized and reflective representation of OAMT patients’ views of QoL and well-being. Furthermore we will use the CERQual approach to assess the confidence in the synthesis findings [46] and we will use the findings with the highest confidence as a reliable representation of patients’ views that could serve to draft items of the initial version of the scale. Pursuing triangulation of data, we will try to gain in comprehensiveness by collecting additional information from truly patient-centered measurement instruments identified from a scoping review.

At the end we will be able to compile a body of evidence to genuinely grasp the perspective from patients on OAMT regarding their perception of well-being and QoL. We firmly consider that such knowledge is likely to lead to greater patient centeredness in considering QoL and well-being in OAMT, which in turn may contribute to a more patient-centered care for opioid-dependent patients in agonist maintenance treatment [60–62].

## Supplementary information

**Additional file 1: Table S1.** Differences between indicators based on hard and soft criteria (adapted from [7]).

**Additional file 2.** PRISMA-P (Preferred Reporting Items for Systematic Review and Meta-analysis Protocols) 2015 checklist for the manuscript: quality of life and well-being from the perspective of patients on opioid agonist maintenance treatment: study protocol for a systematic review of qualitative research and a scoping review of measures.

**Additional file 3.** Strategy designed to search MEDLINE (OVID Medline Epub ahead of print, in-process and other non-indexed citations, Ovid MEDLINE(R) Daily and Ovid MEDLINE(R) 1946 to present).

## Data Availability

Not applicable as no datasets were generated for the development of this protocol. Registration for the systematic review of qualitative research can be checked at the following URL: https://www.crd.york.ac.uk/prospero/display_record.php?RecordID = 86490. The protocol for the scoping review is publicly available at Open Science Framework (https://osf.io/2cks9/).

## Abbreviations

CASP: Critical Appraisal Skills Programme
CERQual: Confidence in the Evidence from Reviews of Qualitative research
COSMIN: COnsensus-based Standards for the selection of health Measurement Instruments
DSM: Diagnostic and Statistical Manual of Mental Disorders
ICD: International Classification of Diseases
OAMT: Opioid agonist maintenance treatment
PRISMA: Preferred Reporting Items for Systematic Reviews and Meta-Analyses
QoL: Quality of life
PROMs: Patient reported outcome measures
