# Supplementary material for: Quality of life and well-being from the perspective of patients on opioid agonist maintenance treatment: study protocol for a systematic review of qualitative research and a scoping review of measures
Source: Syst Rev. 2019 Dec 1;8:299. doi: 10.1186/s13643-019-1237-8 (PMC6886222; doi:10.1186/s13643-019-1237-8)
Supplement: Supplementary file 1 — Additional file 1: Table S1. Differences between indicators based on hard and soft criteria (adapted from [7]). [file 13643_2019_1237_MOESM1_ESM.docx]

**Table 1**. Differences between indicators based on hard and soft criteria (adapted from [7])

| **Hard criteria** | **Soft criteria** |
| --- | --- |
| Efficacious | Effective |
| Third-person knowledge | First-hand knowledge |
| Necessarily assessable | Desirably assessable |
| Based on a negative definition of health | Based on a positive definition of health |
| Centred on the disorder | Patient-centred |
| May arise from the need to justify health care policies | Arises from the need to grasp the patient's perspective |
