# Supplementary material for: Quality of life and well-being from the perspective of patients on opioid agonist maintenance treatment: study protocol for a systematic review of qualitative research and a scoping review of measures
Source: Syst Rev. 2019 Dec 1;8:299. doi: 10.1186/s13643-019-1237-8 (PMC6886222; doi:10.1186/s13643-019-1237-8)
Supplement: Supplementary file 2 — Additional file 2. PRISMA-P (Preferred Reporting Items for Systematic Review and Meta-analysis Protocols) 2015 checklist for the manuscript: quality of life and well-being from the perspective of patients on opioid agonist maintenance treatment: study protocol for a systematic review of qualitative research and a scoping review of measures. [file 13643_2019_1237_MOESM2_ESM.docx]

PRISMA-P (Preferred Reporting Items for Systematic review and Meta-Analysis Protocols) 2015 checklist for the manuscript: QUALITY OF LIFE AND WELL-BEING FROM THE PERSPECTIVE OF PATIENTS ON OPIOID AGONIST MAINTENANCE TREATMENT: STUDY PROTOCOL FOR A SYSTEMATIC REVIEW OF QUALITATIVE RESEARCH AND A SCOPING REVIEW OF MEASURES

| Section and topic | Item No | Location | Checklist item | | |
| --- | --- | --- | --- | --- | --- |
|  | | | | | ADMINISTRATIVE INFORMATION |
| **Title:** |  |  | |  | |
| **Identification** | 1a | p1 line 2 – 5 | | Identify the report as a protocol of a systematic review | |
| **Update** | 1b | N / A | | If the protocol is for an update of a previous systematic review, identify as such | |
| **Registration** | 2 | p2 line 42  p6 line 127 – 128  p9 line 207 – 208 | | If registered, provide the name of the registry (such as PROSPERO) and registration number | |
| **Authors:** |  |  | |  | |
| **Contact** | 3a | P1 line 14 – 30 | | Provide name, institutional affiliation, e-mail address of all protocol authors; provide physical mailing address of corresponding author | |
| **Contributions** | 3b | p16 line 346 | | Describe contributions of protocol authors and identify the guarantor of the review | |
| **Amendments** | 4 | N / A | | If the protocol represents an amendment of a previously completed or published protocol, identify as such and list changes; otherwise, state plan for documenting important protocol amendments | |
| **Support:** |  |  | |  | |
| **Sources** | 5a | p15 line 339 | | Indicate sources of financial or other support for the review | |
| **Sponsor** | 5b | p15 line 339 | | Provide name for the review funder and/or sponsor | |
| **Role of sponsor or funder** | 5c | p15 line 339 | | Describe roles of funder(s), sponsor(s), and/or institution(s), if any, in developing the protocol | |
|  | | | | | INTRODUCTION |
| **Rationale** | 6 | p3 line 58 – 104 | | Describe the rationale for the review in the context of what is already known | |
| **Objectives** | 7 | p5 line 108 – 110 | | Provide an explicit statement of the question(s) the review will address with reference to participants, interventions, comparators, and outcomes (PICO) | |

| Section and topic | Item No | Location | Checklist item |
| --- | --- | --- | --- |
|  | | | METHODS |
| **Eligibility criteria** | 8 | p6 line 131 – 138  p10 line 217 – 220 | Specify the study characteristics (such as PICO, study design, setting, time frame) and report characteristics (such as years considered, language, publication status) to be used as criteria for eligibility for the review |
| **Information sources** | 9 | p7 line 144 – 157  p9 line 213 – 217 | Describe all intended information sources (such as electronic databases, contact with study authors, trial registers or other grey literature sources) with planned dates of coverage |
| **Search strategy** | 10 | p6 line 140 – 148 | Present draft of search strategy to be used for at least one electronic database, including planned limits, such that it could be repeated |
| **Study records:** |  |  |  |
| **Data management** | 11a | p7 line 156  p10 line 225 | Describe the mechanism(s) that will be used to manage records and data throughout the review |
| **Selection process** | 11b | p7 line 156 – 158  p10 line 225 – 227 | State the process that will be used for selecting studies (such as two independent reviewers) through each phase of the review (that is, screening, eligibility and inclusion in meta-analysis) |
| **Data collection process** | 11c | p7 line 170 – 174  p10 line 228 – 240 | Describe planned method of extracting data from reports (such as piloting forms, done independently, in duplicate), any processes for obtaining and confirming data from investigators |
| **Data items** | 12 | N / A | List and define all variables for which data will be sought (such as PICO items, funding sources), any pre-planned data assumptions and simplifications |
| **Outcomes and prioritization** | 13 | N / A | List and define all outcomes for which data will be sought, including prioritization of main and additional outcomes, with rationale |
| **Risk of bias in individual studies** | 14 | p7 line 161 – 169  p10 line 230 – 235 | Describe anticipated methods for assessing risk of bias of individual studies, including whether this will be done at the outcome or study level, or both; state how this information will be used in data synthesis |
| **Data synthesis** | 15a | p8 line 179 – 182  p10 line 243 – 246 | Describe criteria under which study data will be quantitatively synthesised |
|  | 15b | N / A | If data are appropriate for quantitative synthesis, describe planned summary measures, methods of handling data and methods of combining data from studies, including any planned exploration of consistency (such as I^2^, Kendall’s τ) |
|  | 15c | N / A | Describe any proposed additional analyses (such as sensitivity or subgroup analyses, meta-regression) |
|  | 15d | p8 line 182 – 186  p10 line 243 – 246 | If quantitative synthesis is not appropriate, describe the type of summary planned |
| **Meta-bias(es)** | 16 | N / A | Specify any planned assessment of meta-bias(es) (such as publication bias across studies, selective reporting within studies) |
| **Confidence in cumulative evidence** | 17 | p8 line 189 – 200 | Describe how the strength of the body of evidence will be assessed (such as GRADE) |

*From: Shamseer L, Moher D, Clarke M, Ghersi D, Liberati A, Petticrew M, Shekelle P, Stewart L, PRISMA-P Group. Preferred reporting items for systematic review and meta-analysis protocols (PRISMA-P) 2015: elaboration and explanation. BMJ. 2015 Jan 2;349(jan02 1):g7647.*

*The copyright for PRISMA-P (including checklist) is held by the PRISMA-P Group and is distributed under a Creative Commons Attribution Licence 4.0.*
