# Supplementary material for: Quality of life and well-being from the perspective of patients on opioid agonist maintenance treatment: study protocol for a systematic review of qualitative research and a scoping review of measures
Source: Syst Rev. 2019 Dec 1;8:299. doi: 10.1186/s13643-019-1237-8 (PMC6886222; doi:10.1186/s13643-019-1237-8)
Supplement: Supplementary file 3 — Additional file 3. Strategy designed to search MEDLINE (OVID Medline Epub ahead of print, in-process and other non-indexed citations, Ovid MEDLINE(R) Daily and Ovid MEDLINE(R) 1946 to present). [file 13643_2019_1237_MOESM3_ESM.docx]

**Strategy designed to search MEDLINE** (OVID Medline Epub Ahead of Print, In-Process & Other Non-Indexed Citations, Ovid MEDLINE(R) Daily and Ovid MEDLINE(R) 1946 to Present)

1. Search strategy for the scoping review

1 exp Substance-Related Disorders/

2 ((drug* or substance*) adj5 (dependen* or abuse* or addict* or use*)).ti.

3 addiction.ti.

4 exp METHADONE/

5 exp BUPRENORPHINE/

6 opioid*.ti,ab.

7 opiate*.ti,ab.

8 heroin.ti,ab.

9 ((injection or injecting) adj3 (drug* or opioid* or opiate*)).ti,ab. (15603)

10 IDU*.ti,ab.

11 opioid dependen*.ti,ab.

12 opioid substitution.ti,ab.

13 heroin dependen*.ti,ab.

14 opiate dependen*.ti,ab.

15 buprenorphine.ti,ab.

16 suboxone.ti,ab.

17 LAAM.ti,ab.

18 opioid maintenance.ti,ab.

19 opiate maintenance.ti,ab.

20 opioid-agonist*.ti,ab.

21 maintenance therap*.ti,ab.

22 maintenance treatment.ti,ab.

23 heroin maintenance.ti,ab.

24 morphine maintenance.ti,ab.

25 1 or 2 or 3 or 4 or 5 or 6 or 7 or 8 or 9 or 10 or 11 or 12 or 13 or 14 or 15 or 16 or 17 or 18 or 19 or 20 or 21 or 22 or 23 or 24

26 exp "Quality of Life"/

27 quality of life.ti,ab.

28 qol.ti,ab.

29 well being.ti,ab.

30 wellbeing.ti,ab.

31 health status.ti,ab.

32 26 or 27 or 28 or 29 or 30 or 31

33 25 and 32

34 exp PSYCHOMETRICS/

35 exp Patient Health Questionnaire/

36 exp Behavior Rating Scale/

37 exp Patient Reported Outcome Measures/

38 exp Validation Studies/

39 questionnaire*.ti,ab.

40 psychometr*.ti,ab.

41 measure.ti,ab.

42 measures.ti,ab.

43 instrument*.ti,ab.

44 tool.ti,ab.

45 tools.ti,ab.

46 item.ti,ab.

47 items.ti,ab.

48 scale.ti,ab.

49 scales.ti,ab.

50 subscale*.ti,ab.

51 validation.ti,ab.

52 validity.ti,ab.

53 reliability.ti,ab.

54 internal consistency.ti,ab.

55 convergent.ti,ab.

56 discrimina*.ti,ab.

57 construct.ti,ab.

58 34 or 35 or 36 or 37 or 38 or 39 or 40 or 41 or 42 or 43 or 44 or 45 or 46 or 47 or 48 or 49 or 50 or 51 or 52 or 53 or 54 or 55 or 56 or 57

59 33 and 58

1. Search strategy for the synthesis of qualitative research

1 exp Substance-Related Disorders/

2 ((drug* or substance*) adj5 (dependen* or abuse* or addict* or use*)).ti.

3 addiction.ti.

4 exp METHADONE/

5 exp BUPRENORPHINE/

6 opioid*.ti,ab.

7 opiate*.ti,ab.

8 heroin.ti,ab.

9 ((injection or injecting) adj3 (drug* or opioid* or opiate*)).ti,ab. (15603)

10 IDU*.ti,ab.

11 opioid dependen*.ti,ab.

12 opioid substitution.ti,ab.

13 heroin dependen*.ti,ab.

14 opiate dependen*.ti,ab.

15 buprenorphine.ti,ab.

16 suboxone.ti,ab.

17 LAAM.ti,ab.

18 opioid maintenance.ti,ab.

19 opiate maintenance.ti,ab.

20 opioid-agonist*.ti,ab.

21 maintenance therap*.ti,ab.

22 maintenance treatment.ti,ab.

23 heroin maintenance.ti,ab.

24 morphine maintenance.ti,ab.

25 1 or 2 or 3 or 4 or 5 or 6 or 7 or 8 or 9 or 10 or 11 or 12 or 13 or 14 or 15 or 16 or 17 or 18 or 19 or 20 or 21 or 22 or 23 or 24

26 exp "Quality of Life"/

27 quality of life.ti,ab.

28 qol.ti,ab.

29 health state.ti,ab.

30 health status.ti,ab.

31 feel*.ti,ab.

32 well being.ti,ab.

33 wellbeing.ti,ab.

34 recover*.ti,ab.

35 meaning*.ti,ab.

36 26 or 27 or 28 or 29 or 30 or 31 or 32 or 33 or 34 or 35

37 25 and 36

38 exp Qualitative Research/

39 exp Focus Groups/

40 exp NARRATION/

41 exp Interviews as Topic/

42 qualitative.ti,ab.

43 ethnograph*.ti,ab.

44 grounded.ti,ab.

45 focus group*.ti,ab.

46 indepth.ti,ab.

47 fieldwork.ti,ab.

48 field work.ti,ab.

49 field stud*.ti,ab.

50 field research.ti,ab.

51 interview*.ti,ab.

52 purposive*.ti,ab.

53 theoretical.ti,ab.

54 key informant.ti,ab.

55 participant observ*.ti,ab.

56 experience*.ti,ab.

57 theme*.ti,ab.

58 thematic.ti,ab.

59 indepth.ti,ab.

60 38 or 39 or 40 or 41 or 42 or 43 or 44 or 45 or 46 or 47 or 48 or 49 or 50 or 51 or 52 or 53 or 54 or 55 or 56 or 57 or 58 or 59

61 37 and 60
